# Point‐of‐Care Blood Eosinophils to Predict Preschool Wheeze Attacks

**DOI:** 10.1111/all.16500

**Published:** 2025-02-20

**Authors:** Kushalinii Hillson, Sara Fontanella, Hernani Almeida, Barbara Pavlou, Katariina Lajunen, Samantha Irving, Ilaria Testa, Yvonne Bingham, Karina Mayoral Oritz, Shane Lacbay, Sophie Hay, Mindy Gore, Elizabeth Scotney, Emmanouil Paraskakis, Samatha Sonnappa, Louise Fleming, Andrew Bush, Sejal Saglani

**Affiliations:** ^1^ National Heart & Lung Institute Imperial College London London UK; ^2^ Department of Respiratory Paediatrics Royal Brompton Hospital London UK; ^3^ Department of Paediatrics and Adolescent Medicine Turku University Hospital and University of Turku, Lighthouse Hospital Turku Finland; ^4^ Department of Paediatrics Heraklion University Hospital, University of Crete Medical School Crete Greece; ^5^ Centre for Paediatrics & Child Health Imperial College London London UK

**Keywords:** biomarker, eosinophils, machine learning, point‐of‐care test, preschool wheeze, risk factor, wheeze attacks

## Abstract

**Background:**

Post hoc analysis of clinical trials shows blood eosinophil counts (BEC) predict future preschool wheeze attacks; however, prospective usefulness in a clinical setting is unreported. We assessed the feasibility of point‐of‐care (POC) eosinophil measurements in preschool wheezers and related BEC to symptoms, lung function, and utility in predicting attacks.

**Methods:**

Children aged 1–5 years with recurrent wheeze underwent finger‐prick sampling during the outpatient clinic for POC eosinophils, forced oscillation technique (FOT) and/or spirometry, and symptom score (TRACK questionnaire). The utility of BEC and/or the other tests in predicting wheeze attacks in the subsequent 3 months was analysed by comparing those with and without an attack and using a predictive decision tree (DT) model.

**Results:**

Seventy‐three children (median age 4.27 years) were recruited; BEC were higher in atopic children (median 0.5 × 10^9^/L vs. 0.3 × 10^9^/L non‐atopic, *p* < 0.01). BEC moderately correlated with FOT reactance bronchodilator reversibility *z*‐score changes (*r* = 0.495, *p* = 0.005), but no other lung function measures or TRACK score.

68/73 (93%) children were followed up at 3 months. 29/68 (43%) children had > 1 wheeze attack requiring unscheduled healthcare attendance. Absolute and %eosinophils at the baseline visit were higher in those who had an attack (median 0.5 × 10^9^/L vs. 0.3 × 10^9^/L, *p* = 0.03 and median 6% vs. 4%, *p* < 0.01). The DT model showed children with BEC ≥ 4% and TRACK score < 75 were more likely to have a future attack (probability 0.63).

**Conclusion:**

POC blood eosinophils were feasible in a clinical setting. Our preliminary data suggest elevated BEC with a low symptom score predicts a wheeze attack within 3 months.

## Graphical abstract

Absolute and % BEC at baseline are higher in children who had > 1 wheeze attack requiring unscheduled healthcare attendance in follow‐up period. Decision tree model shows children with BEC ≥ 4% and TRACK score < 75 are more likely to have a future attack. Preliminary data suggest elevated BEC with a low symptom score predicts a wheeze attack within 3 months.

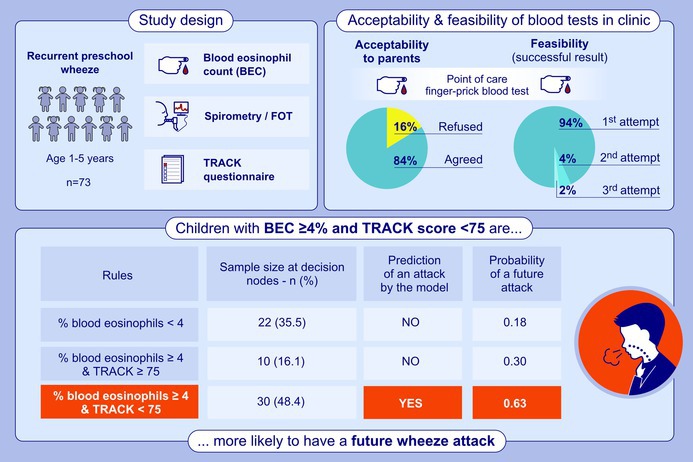

## 1 Introduction

There are few objective markers that can be pragmatically measured in the outpatient setting to identify preschool children who are at risk of future wheeze attacks [1]. Raised blood eosinophil counts (BEC) in adults with mild asthma were associated with a history of previous asthma attacks requiring treatment with oral corticosteroids (OCS) [2]. In preschool children, raised BEC (and evidence of aeroallergen sensitisation) identified children likely to respond better to inhaled corticosteroids (ICS) maintenance therapy to reduce exacerbations [3]. Post hoc analysis of three clinical trials in preschool wheezers (*n* = 1074) showed that when a higher BEC cut‐point was used (0.3 × 10^9^/L compared to 0.15 × 10^9^/L), the odds of a wheeze attack and more frequent hospitalisations were higher [1]. However, the BEC results were obtained by blood sampling using venepuncture and were used to provide the risk of annualised exacerbation rate.

BEC in preschool children, during health, is higher than in older children and adults [4]. Normal values reduce with age, plateauing around puberty and remaining stable in adulthood [4]. This makes it important not to extrapolate from adult data [5, 6]. The current accepted threshold of using blood eosinophilia to guide treatment in preschool wheezers is ≥ 0.3 × 10^9^/L, based on data from the Individualised Therapy for Asthma in Toddlers (INFANT) trial, which showed this was the cut‐off for differential responders to ICS [3]. However, to identify children at high risk of an attack in the clinic, it is important to have results available immediately at the time of review, in a similar way to lung function and symptom scores, to enable management changes. Use of point‐of‐care (POC) blood eosinophil assessments, measured from a finger‐prick capillary blood sample, can potentially achieve this [7]. However, to date, there are no publications that report the feasibility or acceptability of this approach in children, nor the added value of BEC as a biomarker in the management of preschool wheeze. The utility of BEC as an objective biomarker in preschool children remains unknown.

We hypothesised that finger‐prick blood tests would be acceptable to parents and children, thus making it feasible when undertaken using a POC device in a tertiary clinic setting. We also hypothesised that elevated BEC would identify preschool children at higher risk of a future wheeze attack. The aims of our study were to investigate: (1) The feasibility and acceptability of undertaking POC blood eosinophil measurements in preschool children with recurrent wheeze in a real‐life clinic setting, (2) the relationship of BEC with other tests including spirometry, forced oscillation technique (FOT) and symptom scores, and (3) the utility of BEC as an objective marker either alone or in combination with symptom score and/or lung function in predicting future wheeze attacks in preschool children by the time of the next clinic appointment.

## 2 Methods

### 2.1 Study Design

A single‐centre prospective study undertaken at a specialist paediatric respiratory clinic over two periods (February 2022–May 2023 and October 2023–March 2024). All clinical assessments, blood, and lung function tests were undertaken on the same day with informed parental consent. This study was granted approval by the London Hampstead Research Ethics Committee (REC number 15/LO/1885).

### 2.2 Participants

Children aged between 1–5 years, with a diagnosis of recurrent wheeze, were recruited [6]. Recurrent wheeze was defined as more than one attack of doctor‐diagnosed preschool wheeze ever. Those with a known alternative chronic lung disease such as cystic fibrosis or bronchiectasis were not eligible. Participant demographics including age, sex, ethnicity, weight, height, body mass index (BMI), gestational age at birth and prescribed medications were recorded. Symptom control was assessed using the Test for Respiratory and Asthma Control (TRACK) score [8, 9] which is a validated questionnaire administered to parents or carers and is used to measure asthma or wheeze burden and symptom control in young children aged between 1–5 years [8, 9, 10] (Figure S1). A score of ≥ 80/100 is classified as good symptom control, and a change in score of 10 is considered a minimal clinically important difference (MCID) [10]. The number of wheeze attacks that had required unscheduled healthcare attendances (emergency department [ED] attendance or hospitalisation for wheeze) in the previous 6 months was collected from participants' electronic patient records or parental reports. Atopic status was defined as at least one positive skin prick test or raised specific IgE to a standard panel of six aeroallergens (house dust mite, cat, dog, grass pollen, tree pollen and aspergillus).

### 2.3 Blood Eosinophils

Children underwent a finger‐prick blood test using a Sarstedt neonatal safety lancet, and MicroCuvette (HemoCue) was used to collect capillary blood samples. The blood sample was analysed using the validated HemoCue WBC Diff Systems device [11, 12]. Total white cell count was obtained, with a differential cell count (×10^9^L) and % cell count. The results generated by the POC device were validated against laboratory venous blood tests in children who had this test for clinical indications. The feasibility of the POC blood test was assessed by the number of pricks of the finger attempted to obtain an adequate sample for a valid result in the HemoCue. An error result was recorded if no valid result was obtained or after a maximum of three finger‐pricks.

### 2.4 Acceptability of Blood Eosinophil Test

Acceptability of the finger‐prick blood test was assessed using validated LIKERT scale questionnaires consisting of two questions establishing the child's and parent/carer's perspective on the blood test as a POC and as a routine test [13]. The LIKERT scale given to patients consisted of two faces (happy and sad) (Figure S2A). Parents/carers' LIKERT scale included five options ranging from: ‘strongly agree’ to ‘strongly disagree’ for each question (Figure S2B) [14].

### 2.5 Lung Function Tests

Lung function was assessed using the forced oscillation technique (FOT) using Resmon Pro (Restech, Minnesota, USA). Post‐bronchodilator percentage change to resistance (Rtot %) change [15] and *Z*‐score change to reactance (Xtot change) were measured using the 8 Hz paediatric protocol, and reference values used were based on Calogero et al. (*z* score change of −1.83 for Rtot and 1.95 for Xtot) [15]. Children also attempted spirometry, measured using Vitalograph Alphatouch (Vitalograph, Buckingham, UK), undertaken according to the American Thoracic Society/European Respiratory Society (ATS/ERS) guidelines [16, 17]. FEV_1_% predicted, FEV_1_/FVC ratio, and bronchodilator reversibility (BDR%) following 400 mcg salbutamol were recorded and related to BEC.

### 2.6 Follow‐Up Data

The follow‐up period was defined as the time interval between the baseline clinic visit, when the POC blood test was done, and the child's next clinic appointment. A follow‐up verbal questionnaire was administered for those seen during their clinic appointment to ascertain the number of wheeze attacks during this time. In children whose parents did not have time for the questionnaire during their appointment, clinic letters from the visit following the POC blood test were used to quantify the number of wheeze attacks during this time period. In children who did not have a clinic appointment during the follow‐up period, the verbal questionnaire was administered over the telephone.

### 2.7 Statistical Analysis

A sample size calculation was not undertaken as this was a pilot study and we are not aware of previous studies that have investigated the utility of POC blood eosinophils prospectively in a clinical setting. The agreement between the POC device BEC results and the gold standard laboratory venous BEC was evaluated using the Bland–Altman analysis and intraclass correlation coefficient (ICC). Associations between continuous variables were analysed using the Spearman's correlation coefficient. Comparisons between groups were undertaken using the nonparametric Mann Whitney *U* test. Data were analysed using Graphpad Prism v10. *p* < 0.05 was accepted as statistically significant. ICC and ROC curve analysis were performed using R to analyse the optimum cut‐off of blood eosinophil absolute count and eosinophil percentage as objective predictors of wheeze exacerbations.

We used a supervised machine learning model to explore whether BEC combined with symptom score and/or atopic status and/or blood neutrophils is more accurate at predicting future attacks than BEC alone. To this end, we used a decision tree model, a nonparametric supervised learning method frequently used for classification and regression problems [18]. Decision trees learn simple decision rules from data features to predict target values. They have a structure akin to a flowchart. Internal nodes represent attribute tests, branches indicate test outcomes, and leaf nodes hold class labels. Decision tree analyses can also work with many forms of data, such as mixed data, missing values and outliers, and due to their simplicity in comprehension and interpretation, are frequently used in healthcare applications. In our analysis, input variables for the classification models were BEC and neutrophils (both percentage and absolute count), atopy (measured as presence\absence) and symptom scores (TRACK). These parameters were chosen as they had the most complete data for all patients. The model was trained using 10‐fold cross‐validation repeated 10 times, and Gini's Diversity Index (GDI) served as the splitting criterion to measure node impurity. Given the small sample size and the exploratory nature of the analysis, we did not divide the data into training and test sets.

## 3 Results

Eighty‐two children with recurrent preschool wheeze were recruited for this study; 9 children were excluded from analysis as BEC was not obtained. The reasons for this were parental time limitations meaning the test was not attempted (*n* = 8) or finger‐prick blood sampling was unsuccessful (*n* = 1). Seventy‐three children with a BEC result were included in the analysis, and the demographics of the included and excluded children (with no obvious differences between the groups) are summarised in Table 1.

**TABLE 1:** Summary of the demographics of the 73 children included, and 9 excluded at baseline.

	Included *n* = 73	Excluded *n* = 9
Age, years	4.27 (2.0–5.9)	5.2 (2.6–6)
Sex		
M	50 (68.5%)	6 (66.6%)
F	23 (31.5%)	3 (33.3%)
Height, cm	103.3 (79–125.2)	106.8 (92.5–114.0)
Weight, kg	16.35 (11–24.8)	16.0 (12.6–21.1)
BMI kg/m^2^	15.7 (11.8–19.5)	14.7 (13.5–16.3)
Ethnicity (GLI)		
Caucasian	54 (74%)	4 (44.4%)
Black	6 (8.2%)	1 (11.1%)
Northeast Asian	2 (2.7%)	0
Southeast Asian	0 (0%)	0
Other	11 (15.1%)	4 (44.4%)
Gestational age		
≥ 36 weeks	61 (83.6%)	7 (77.8%)
< 36 weeks	12 (16.4%)	2 (22.2%)
Atopic	37 (50.7%)	6 (66.7%)
Other related comorbidities		
Eczema	10 (13.7%)	2 (22.2%)
Allergic rhinitis	9 (12.3%)	0
Medication prescription		
SABA	66 (90.4%)	8 (88.9%)
ICS	42 (57.5%)	8 (88.9%)
ICS + LABA	22 (30.1%)	0
Montelukast	21 (28.8%)	0
Oral prednisolone	2 (2.7%)	0
LAMA	2 (2.7%)	0
Antihistamine	4 (5.5%)	0
Budesonide dose/day (mcg)	400 (100–1000)	400 (100–500)
≥ 400 BDP equivalent per day	44 (60.3%)	5 (55.6%)
< 400 BDP equivalent per day	20 (27.4%)	3 (33.3%)
Not on ICS	9 (12.3%)	0
Exacerbations in last 6 months		
Requiring unscheduled healthcare visit	*n* = 42 (57.5%)	
Requiring admission to hospital	*n* = 33 (45.2%)	
Total white cell count (×10^9^L)	7.6 (3.6–13.8)	
Eosinophil count (×10^9^L)	0.3 (0.0–1.6)	
Eosinophil %	5 (0–14)	
Lymphocyte count (×10^9^L)	3.6 (1.4–7.4)	
Lymphocyte %	49 (13–68)	
Neutrophil count (×10^9^L)	2.8 (1.0–11.5)	
Neutrophil %	39 (21–77)	
Monocyte count (×10^9^L)	0.5 (0.2–1.2)	
Monocyte %	7 (3–12)	
Basophil count (×10^9^L)	0 (0–0.1)	
Basophil %	0	
Spirometry; *n* = 26 (35.6%)		
FEV_1_ (L)	1.02 (0.53–1.41)	
FEV_1_ %Predicted	88 (57–118)	
FEV_1_/FVC	0.93 (0.64–1.15)	
FOT (8 Hz); *n* = 34 (46.6%)		
Rtot change (z score)	−1.03 (−7.36–2.28)	
Xtot change (z score)	1.36 (−1.69–7.21)	
TRACK score; *n* = 62	55 (10–100)	
Score ≥ 80 (good symptom control), *n* (%)	13/73 (17.8%)	
Score < 80 (poor symptom control), *n* (%)	49/73 (67.1%)	

*Note:* Data represented as median (range) or number of participants (%).

Abbreviations: %, percentage; Atopic, parental reporting of confirmed aeroallergen or food sensitisation, confirmed either on specific IgE or skin prick testing; BDP, beclometasone dipropionate; BMI, body mass index; cm, centimetres; F, female; FEV1, forced expiratory volume in 1 s; FOT, forced oscillation technique; FVC, forced vital capacity; GLI, global lung initiative; ICS, inhaled corticosteroids; kg, kilogrammes; LABA, long‐acting β agonist; LAMA, long‐acting muscarinic antagonists; M, male; mcg, micrograms; n, number; Rtot, total post‐bronchodilator change in resistance; SABA, short‐acting beta agonist; x᷉, median; Xtot change, total post‐bronchodilator change in reactance.

### 3.1 Acceptability and Feasibility of Finger‐Prick Blood Tests in Clinic

Acceptability and feasibility data of the finger‐prick test was available for 67 children. Children and parents/carers were approached in the clinic and 56/67 (84%) agreed to have the POC test, with the main reason for refusal being needle anxiety. Of those who had the test, 98% of parents/carers and 81% of children found the POC test acceptable, and most were happy to have the test performed routinely (Table 2). A successful result was obtained in 94% of children with one attempt, with only two requiring more than one attempt and a failure in one child.

**TABLE 2:** Acceptability and feasibility of finger‐prick blood eosinophil test in children with wheeze and asthma (*n* = 67).

Number of children whose parents/carers were approached	67
Number of children whose parents/carers agreed to participate	56/67 (84%)
Number of children who had the test (8 had to leave clinic before test was done)	48/56 (86%)
Age (years); median (range)	4.3 (1–5)
Median child rating score: How they found it^a^	2
Median parent rating score: Acceptable method to obtain blood sample^b^	5 (98% scored 5)
Median parent rating score: Acceptable for child to have this done routinely^b^	5 (81% scored 5)
Successful sample obtained first attempt (out of 48)	45 (94%)
Successful sample obtained > 1 attempt (out of 48)	2 (4%)
Finger‐prick blood eosinophil count (10^9^/L); median (range)	0.40 (0–3.8)

^a^ Children rated score from a 2‐point smiley Likert scale (1—sad face; 2—happy face).

^b^ Parent rated score from a 5‐point Likert scale (1—worst; 5—best).

### 3.2 Validation of the HemoCue WBC Diff Systems Device

In 14 children who had clinically indicated laboratory processed venepuncture BEC, a capillary BEC was done at the same time to validate the POC device results. There was a very high agreement between the POC capillary BECs compared to the venepuncture sample processed in the lab, with an intraclass correlation coefficient (ICC) of 0.978 and the Bland–Altman analysis showing very good agreement between the two tests (Figure S3).

### 3.3 Relationship Between Blood Eosinophils and Baseline Demographics

**FIGURE 1:**
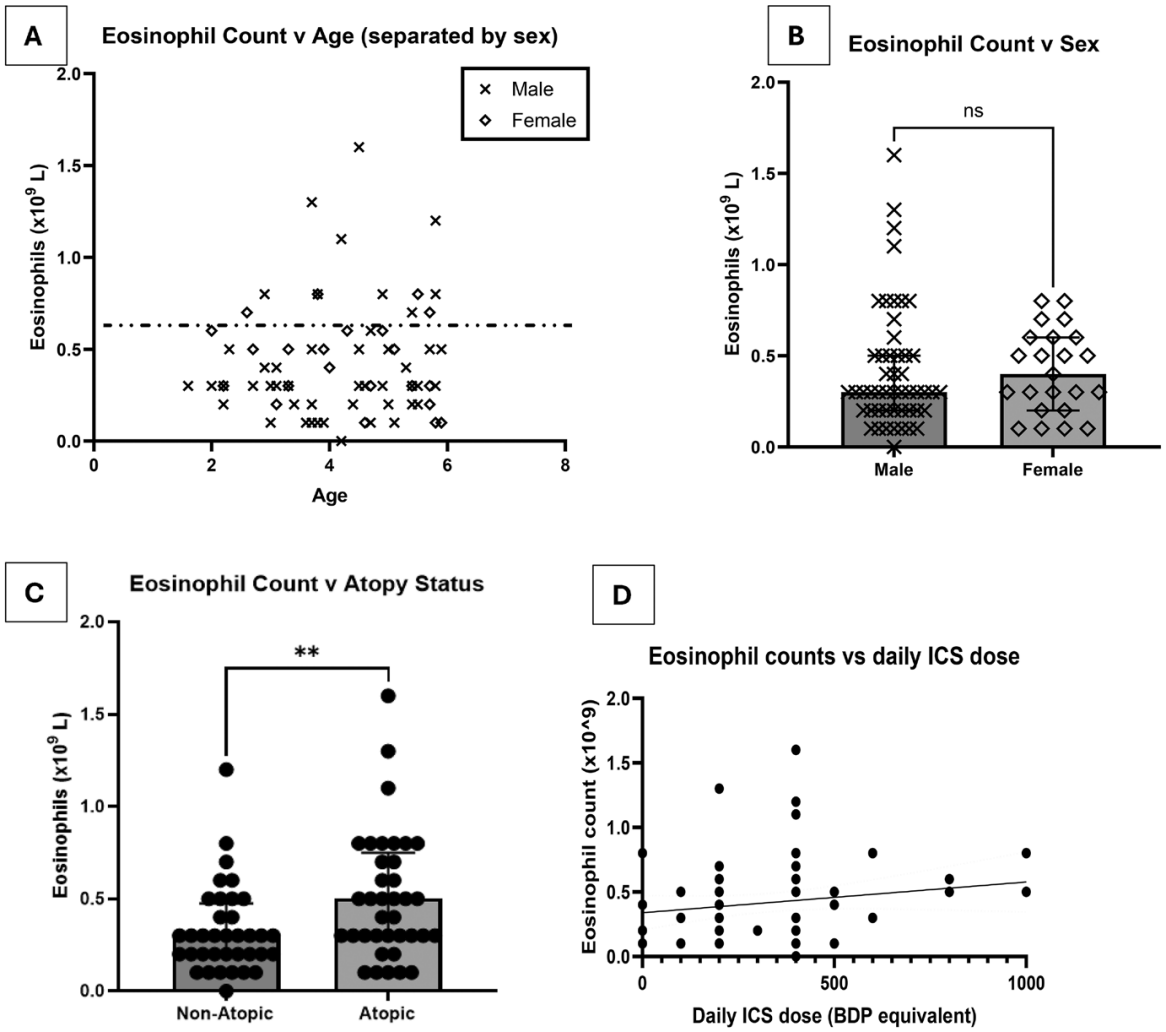
Comparison of eosinophil counts between (B) males and females, (C) atopic and non‐atopic children, and (D) relationship between blood eosinophil counts and daily ICS dose (BDP equivalent). (A) Distribution of eosinophil count by age, with male and female patients indicated. Spearman's correlation *r* = 0.033; *p* = 0.78; *n* = 73 (B) Median eosinophil counts for males (*n* = 50) were 0.3 × 10^9^/L and 0.4 × 10^9^/L for females (*n* = 23) (male IQR = 0.2 × 10^9^/L – 0.5 × 10^9^/L; female = 0.2 × 10^9^/L – 0.6 × 10^9^/L). *p* value = 0.5724 (C) Median eosinophil count in atopic wheeze 0.5 × 10^9^/L (IQR 0.3 × 10^9^/L − 0.8 × 10^9^/L) and non‐atopic wheeze median 0.3 × 10^9^/L (IQR 0.2 × 10^9^/L – 0.5 × 10^9^/L). *p* = 0.0089. *n* = 73. (D) Correlation between blood eosinophil count and daily ICS dose (*r* = −0.23, *p* = 0.05, *n* = 73). Non‐parametric Mann–Whitney *U* Test used. ns, not significant, **p < 0.01. Non‐parametric Spearman's correlation was performed for correlation. Error bars show interquartile range. Graph bar shows median. Black line shows linear regression with 95% confidence interval boundaries. Data points represent individual study participants.

In the 73 children with a valid POC test result, the median (interquartile range) BEC were 0.3 × 10^9^/L (0.2 × 10^9^/L – 0.5 × 10^9^/L). There was no relationship between BEC and either age or sex (Figure 1A,B). An almost equal distribution of children who were atopic (*n* = 37) and non‐atopic (*n* = 36) was recruited. Atopic preschool wheezers had significantly higher BEC (median 0.5 × 10^9^/L [IQR 0.3 × 10^9^/L – 0.8 × 10^9^ L]) compared to non‐atopic wheezers, although there was considerable overlap between the groups (median 0.3 × 10^9^/L [IQR 0.2 × 10^9^/L – 0.5 × 10^9^/L]) (Figure 1C).

In the study, 64/73 (88%) preschool wheezers were prescribed ICS, either alone or with long‐acting β agonist (LABA). There was no relationship between prescribed daily ICS dose and BEC (Figure 1D). 34/37 (91.9%) of atopic children were prescribed ICS, compared to 30/36 (83.3%) non‐atopic children.

### 3.4 Relationship Between Blood Eosinophils and Wheeze Attacks in the Previous 6 Months

BEC in children at the clinic was not significantly different between those who had at least one attack in the previous 6 months and those who had no wheeze attacks (Figure S4A). There was also no significant difference in BEC between children with normal (≥ 80) and abnormal (< 80) TRACK scores [8, 9] (Figure S4B).

### 3.5 Relationship Between Blood Eosinophils and Lung Function

Measuring lung function is challenging in this age group, with only 26 of the 73 (35.6%) preschool children able to complete spirometry, compared to 34 children (46.6%) who were able to perform FOT. The children who were only able to perform FOT were significantly younger than those who performed spirometry and FOT (Figure S5A). There was no difference in BEC between children with normal FEV1 (≥ 80% predicted) and abnormal FEV1 (< 80% predicted) (Figure S5B). No correlation was observed between BEC and FEV1 or FEV1% predicted (Figures S5C and S5D).

Using FOT measurements, there was a moderate correlation between Xtot bronchodilator reversibility *z* score change (*r* = 0.495, *p* = 0.005), which is a measurement of airway reactance and blood eosinophil counts (Figure S6A). However, a correlation was not observed between Rtot bronchodilator reversibility *z* score change, which is a measure of airway resistance and BEC (Figure S6B).

### 3.6 Blood Eosinophils, Symptom Score and Lung Function as Predictors for Future Wheeze Attacks

**FIGURE 2:**
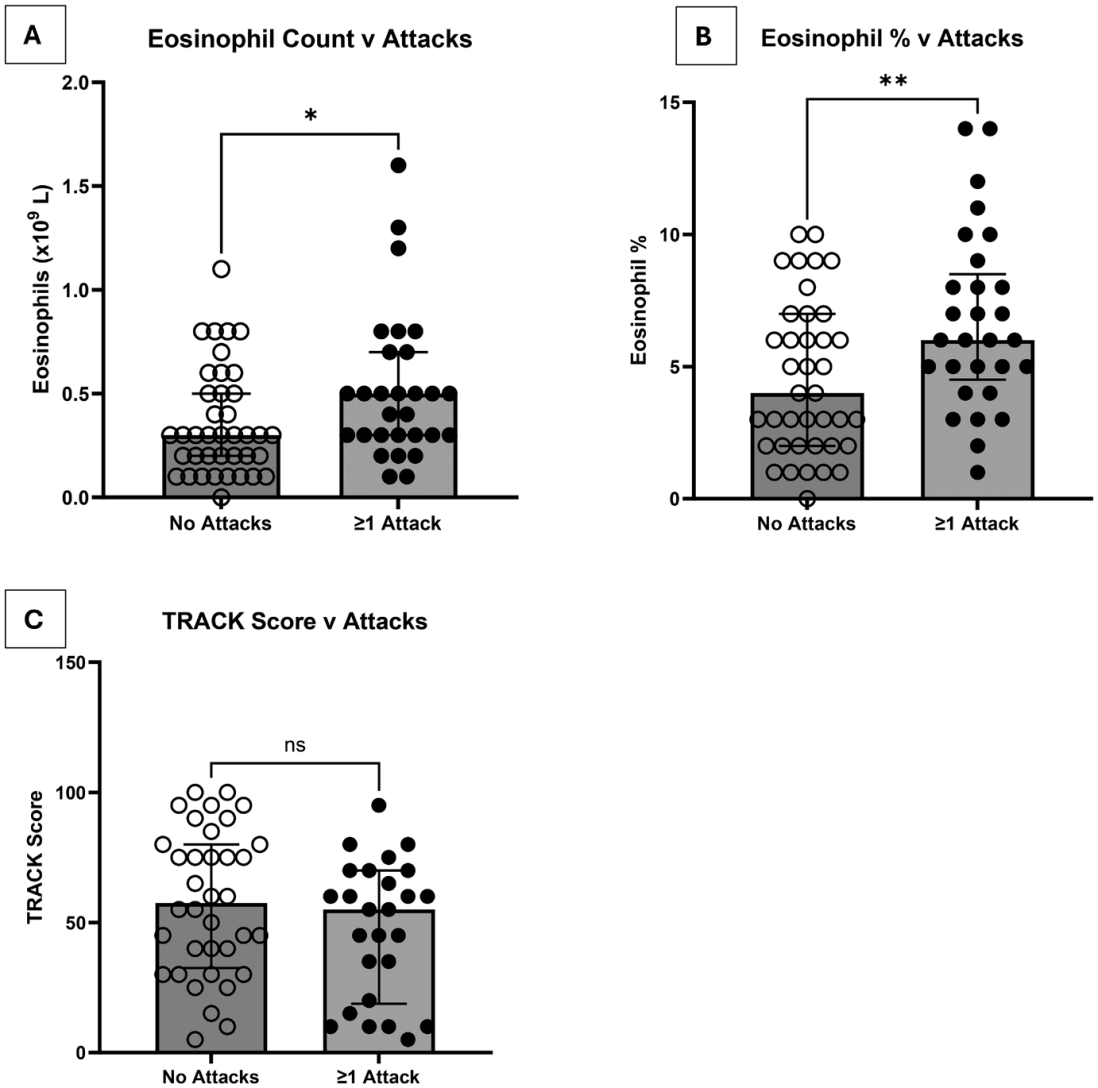
(A) Blood eosinophil absolute count and (B) blood eosinophil percentage in children with and without future attacks. (C) Comparison between TRACK score at baseline and future attacks. (A) Median eosinophil count in children with no exacerbations (*n* = 39) 0.3 × 10^9^/L (IQR: 0.2 × 10^9^/L – 0.5 × 10^9^/L) versus median 0.5 × 10^9^/L (IQR 0.3 × 10^9^/L − 0.7 × 10^9^/L) in children with at least one exacerbation (*n* = 29). *p* = 0.033. (B) Median eosinophil % in children with no exacerbations were 4% (*n* = 39) (IQR 2%–7%) compared to 6% in children who had at least one exacerbation (*n* = 29) (IQR 4%–9%). *p* = 0.009 (C) Median TRACK score for children who had no exacerbations (*n* = 36) was 57.5 (IQR 33–80) compared to a median of 55 in children who then went on to have at least one exacerbation by the next follow‐up (*n* = 26) (19–70). *p* value = 0.18. Mann–Whitney *U* Test was performed. ns, not significant, *p < 0.05, **p < 0.01. Error bars show interquartile range. Graph bar shows median. Individual participant data are plotted as scatter plots.

During the study period, 68/73 children (93.1%) with a BEC result had a follow‐up visit at a median of 3 months (range 1–10 months). The five children who did not have a follow‐up were either discharged from tertiary care or lost to follow‐up. Also, 29/68 (42.6%) had at least one attack that required an unscheduled healthcare visit by the time of the next follow‐up. As a group, children who went on to have at least one wheeze attack requiring unscheduled healthcare attendance had a higher absolute BEC (median 0.5 × 10^9^/L [IQR:0.3 × 10^9^/L − 0.7 × 10^9^/L]) and % blood eosinophils (median 6% [IQR 4%–9%]), compared to those who had no attacks in the subsequent 3 months (absolute count median 0.3 × 10^9^/L [IQR: 0.2 × 10^9^/L – 0.5 × 10^9^/L]) and eosinophil % (median 4% [IQR 2%–7%]). (Figure 2A,B), with considerable overlap between the two groups. ROC curve analyses identified optimum BEC cut‐offs of 0.3 × 10^9^/L for absolute eosinophil counts (area under the curve [AUC] 0.65, accuracy 0.60, sensitivity 0.85, specificity 0.42), and 4% for eosinophil % (AUC 0.70, accuracy 0.65, sensitivity 0.85, specificity 0.5) (Figures S7A, S7B and Table S1).

There was no significant difference in TRACK score (Figure 2C) or any lung function parameters (post bronchodilator resistance change [% change and *z* score change], reactance change [absolute value change and *z* score change] or FEV1% predicted) measured at the baseline visit when comparing children who then went on to have at least one wheeze attack compared to those who did not have an attack.

### 3.7 Development of a Decision Tree Model to Determine Which Measured Parameters Predict Future Wheeze Attacks

**FIGURE 3:**
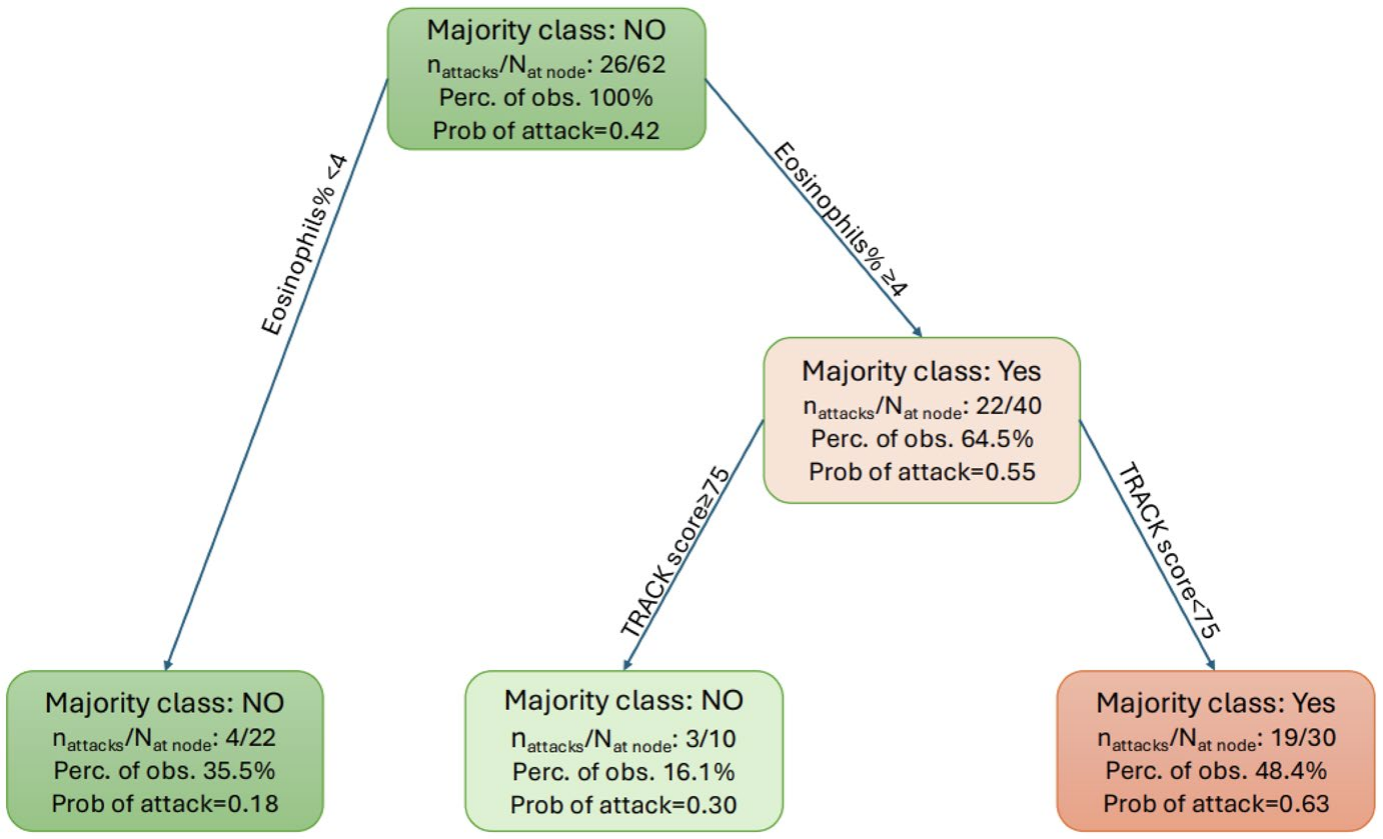
Decision tree model. Decision Tree Model. Clinical variables (eosinophils [absolute count and %], neutrophils [absolute count and %], atopy [0: No and 1: Yes], and symptom scores) were used to generate a decision tree to investigate the utility of additional factors in predicting the risk of an asthma attack in preschool children. In the decision tree, each node represents a decision point based on a clinical variable, and the branches represent the possible outcomes, leading to further nodes or final predictions. Each box displays the predicted class (Attack: Yes or No), the number of children with attacks/total number of children in that node, the percentage of observations at the node, and the probability of a wheeze attack.

In the study, 62/73 (84.9%) children with complete data were included in the decision tree model. Absolute BEC, % eosinophils and neutrophils, and TRACK score were included as continuous variables, and atopy was a binary variable. The model revealed that only some of the variables included were important in predicting future wheeze attacks. Specifically, a combined score with eosinophil percentage > 4% and symptom score (TRACK < 75) together showed an increased probability (0.63) of a wheeze attack in the next 3 months (Figure 3 and Table 3). In contrast, children with blood eosinophils < 4% or those with blood eosinophils ≥ 4% but a TRACK score ≥ 75 were less likely to have a wheeze attack in the subsequent 3 months (probability of an attack of 0.18 and 0.30, respectively) (Table 3). The inclusion of atopic status or blood neutrophils did not provide an improvement in the classification performance. The computed performance measures indicated moderate sensitivity and specificity. The Area Under the Curve (AUC) for BEC and TRACK combined was 0.72, with an accuracy of 0.71, sensitivity of 0.69 and specificity of 0.73, indicating an improved classification performance compared to using a single marker of absolute or % eosinophils (Table S1).

**TABLE 3:** Decision tree model rules for the most important predictors for future wheeze attacks in the subsequent 3 months.

Rules	Sample size at decision nodes—*n* (%)	Prediction of attack by model	Probability of future attack
%blood eosinophils < 4	22 (35.5)	No	0.18
2 %blood eosinophils ≥ 4 and TRACK ≥ 75	10 (16.1)	No	0.30
3 %blood eosinophils ≥ 4 and TRACK < 75	30 (48.4)	Yes	0.63

## 4 Discussion

We have shown POC testing for BEC using a finger‐prick capillary blood sample in a tertiary clinic setting is both feasible and acceptable to preschool children with recurrent wheeze and to their parents/carers. Unbiased analysis using a machine learning approach has enabled us to prospectively demonstrate that higher BEC (≥ 4%), when used in conjunction with symptom score using the TRACK questionnaire (score < 75), may be a useful tool to predict a future wheeze attack within 3 months. We have shown BEC alone was higher in children that had a subsequent attack; however, this was a group finding, with overlap between those that did and did not have an attack. Therefore, our current data suggest BEC alone cannot be used as a biomarker to predict a wheeze attack for the individual child in the setting of a tertiary paediatric referral clinic. This requires validation in an independent and larger cohort.

We have also validated the accuracy of the POC test results against lab‐processed venous samples and shown a very high level of agreement between the two measures. BEC were not influenced by age or sex in this study, which contrasts with previous studies [1, 19, 20]. However, most children included were prescribed maintenance ICS, some in very high doses, which may have masked any age or sex differences that might otherwise be seen in ‘steroid naïve’ children. Also, the numbers are relatively small and a type 2 error (i.e., wrongly state that BEC are helpful in predicting an attack) is possible. Previously published literature from a retrospective post hoc analysis showed that elevated BEC of > 0.3 × 10^9^/L (together with evidence of aeroallergen sensitisation as a second biomarker) was associated with higher odds of exacerbation over 12–18 months in preschool wheeze [1]. However, the data were derived from clinical trials and included children who were prescribed lower dose ICS than our patients. To our knowledge, this is the first study to include an assessment of acceptability and feasibility of a POC test in a real‐life clinic setting in preschool‐aged children with recurrent wheezing. Moreover, all tests were undertaken at the same clinic appointment and related to symptom control and prescribed medication, and recruitment and follow‐up were prospective. We also assessed predictive value for a shorter period, reflective of the frequency of routine clinic appointments.

Even though nearly all the atopic children were prescribed ICS, there was still a relationship between atopic status and elevated BEC, in agreement with previous reports [5]. Accepting that using absolute BEC or % blood eosinophils alone as a predictor confers atopy as being a potential confounding factor, we used a machine learning approach, with unbiased, predictive decision tree modelling, to understand which objective parameters may predict wheeze attacks. Even though atopic status was included in this model, together with blood neutrophils, only % blood eosinophils ≥ 4% together with TRACK score < 75 contributed to the prediction rule for an attack in preschool wheezers.

TRACK score as a second prospective biomarker is useful as it is an easy‐to‐administer, validated questionnaire, which can be routinely used in the clinic setting, without significantly increasing the testing burden for patients. Atopic sensitisation has been identified previously as a second biomarker to add prognostic value to BEC, in terms of stratifying risk for a future attack [1]. However, atopic sensitisation testing is not carried out at every clinic visit in preschool wheezers since atopic status is unlikely to change within the timeframe of 3–6 months. Moreover, once atopic sensitisation to aeroallergens is established, this is unlikely to change, meaning this is not a responsive second biomarker for repeated use. Using TRACK score as a second biomarker may be more useful as it is a dynamic marker that reflects the child's symptom burden and may therefore have greater added value as a prognostic tool when used in conjunction with BEC. However, this needs validation in a second cohort.

It is important to acknowledge certain limitations inherent to the decision tree approach. Firstly, the sample size utilized for training and evaluating the decision tree model was relatively small. While efforts were made to ensure the robustness of the analysis within this sample, the limited size might hinder the generalizability of the model's performance to a larger population. We have not assessed the stability of BEC over time, and only one measurement was taken on a single occasion. Further research is required to establish the longitudinal stability of BEC in preschool children. Secondly, the decision tree model may be improved by exploring a wider range of variables, such as offline fractional exhaled nitric oxide (FeNO). In a previous post hoc analysis in preschool children, looking at the small subset of children who had offline FeNO, there was a reduction in FeNO and improved symptom scores when children were commenced on maintenance ICS [1]. FeNO (≥ 50 ppb) and blood eosinophil counts have been validated to demonstrate airway inflammation in adults with asthma, and have been proposed as a composite score to predict attacks [21]. Therefore, future inclusion of FeNO in a preschool wheeze predictive score may further improve the model's predictive value. We did not include data relating to the duration between the last wheeze episode and BEC in the model as this information would have been affected by recall bias, making the results less reliable.

We have prospectively demonstrated using receiver operator characteristic (ROC) curves that BEC cut‐offs of > 0.3 × 10^9^/L or eosinophil % of ≥ 4%, which are the currently used and accepted values for eosinophilia from previous post hoc analyses [1, 3], have a low specificity (0.5 for eosinophil % and 0.42 for BEC), despite the relatively high sensitivity (0.85 for both), and overall low AUCs (0.7 for eosinophil % and 0.65 for BEC) for both. This supports the suggestion that BEC alone is unlikely to be a useful predictive biomarker for the individual patient, at least in a tertiary referral clinic. Of note, the median BEC for the group who went on to have at least one attack in the subsequent 3 months was 0.5 × 10^9^/L, not 0.3 × 10^9^/L.

We acknowledge one limitation of the HemoCue WBC Diff Systems device is that results are given to one decimal place, while laboratory results are measured to two decimal places. However, this did not impair our ability to define values that predicted a wheeze attack.

We also acknowledge the children included would have more severe wheezing as they were being seen in a specialist respiratory clinic, but these are also the children who are at higher risk of attacks. Therefore, in this high‐risk group, additional objective markers that help to predict attacks are an unmet need. Although most children were already prescribed ICS, the prediction score may alert the clinical team to optimize factors related to the basics of management such as adherence to ICS, inhaler technique, minimizing allergen triggers, and parental education about the need for ICS. If modifiable risk factors have been addressed, a high‐risk score may also highlight children who require therapy escalation.

## 5 Summary

In this pilot and proof‐of‐concept study, we have shown the feasibility and acceptability of measuring POC blood eosinophils in children with recurrent preschool wheeze during a routine tertiary clinic appointment. Our preliminary data suggest blood eosinophils > 4% AND TRACK score < 75 identified children at increased risk of a wheeze attack in the subsequent 3 months, with a probability of 0.63. Children who went on to have an attack, as a group, had higher BEC (≥ 6%, or > 0.5 × 10^9^/L). These findings need to be confirmed in a larger study; however, they pave the way for using POC testing and a future interventional clinical trial to assess the efficacy of biomarker‐driven management of preschool wheeze.

## Author Contributions

K.H. patient recruitment, data analysis and manuscript writing. S.F. data analysis. H.A., B.P., K.L., S.I., I.T., Y.B., K.M.O., S.L., S.H., M.G., E.S. and E.P. patient recruitment. S.S., L.F. and A.B. editing manuscript. S.S. Generation of study design, overall supervision, final editing and writing of manuscript.

## Conflicts of Interest

K Hillson is a contributor to intellectual property licensed by Oxford University Innovation to AstraZeneca, outside the submitted work. Other authors declare no conflicts of interest.

## Supporting information


Data S1.

## Data Availability

The data that support the findings of this study are available from the corresponding author upon reasonable request.
