# Supplementary material for: Point‐of‐Care Blood Eosinophils to Predict Preschool Wheeze Attacks
Source: Allergy. 2025 Feb 20;80(4):1038–46. doi: 10.1111/all.16500 (PMC11969302; doi:10.1111/all.16500)
Supplement: Supplementary file 1 — Data S1. [file ALL-80-1038-s001.docx]

**Supplementary Online Material**

**Supplementary Figure 1 A:** **Test for Respiratory and Asthma Control (TRACK) questionnaire: a caregiver-completed questionnaire for preschool-aged children**


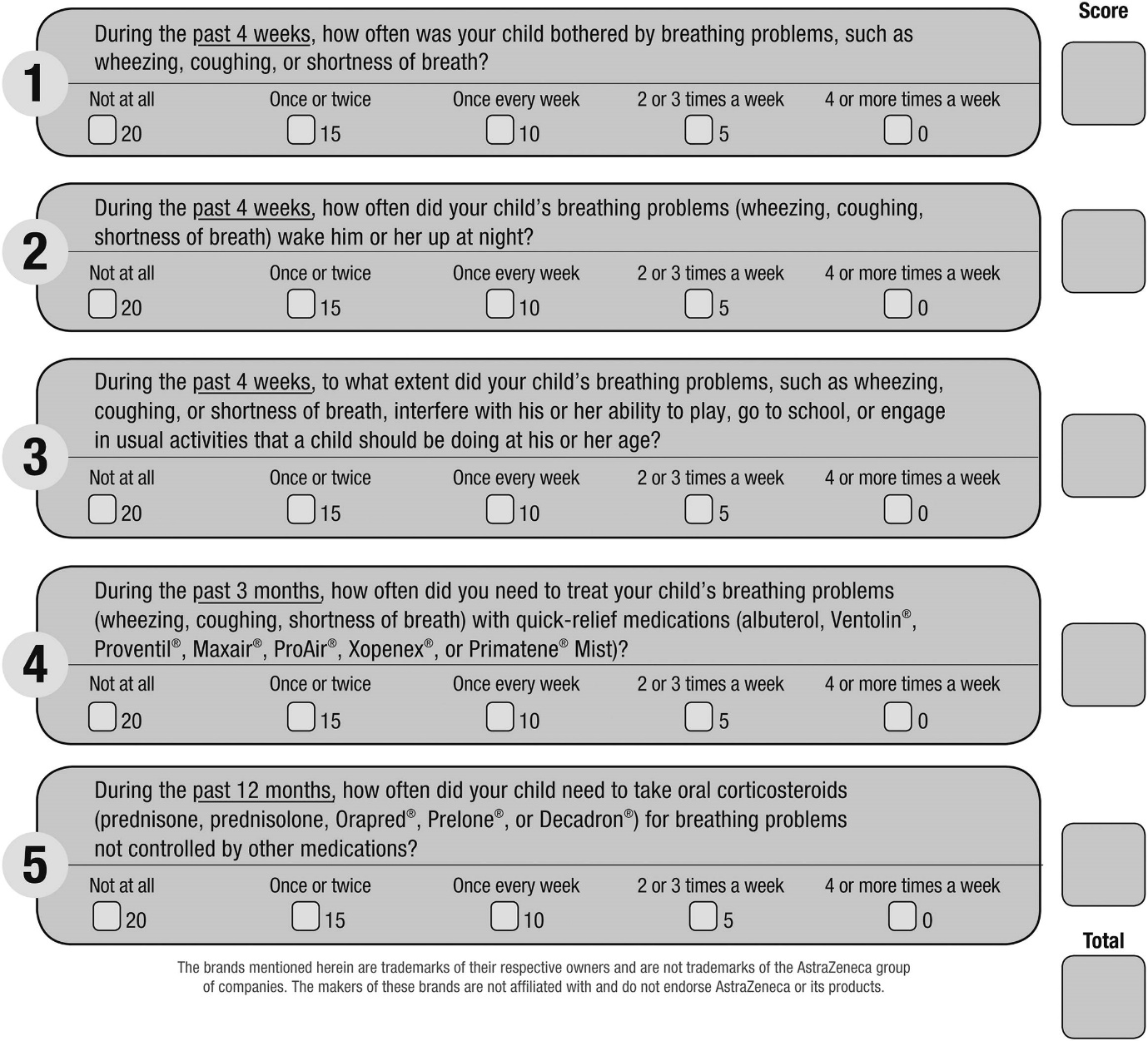


**Supplementary Figure 1 TRACK questionnaire. TRACK is a trademark of the AstraZeneca group of companies.** ©2009 AstraZeneca LP. All rights reserved 278650 5/09. Reprinted with permission from AstraZeneca Pharmaceuticals.

**Supplementary Figure 2A: LIKERT scale given to patients**

**
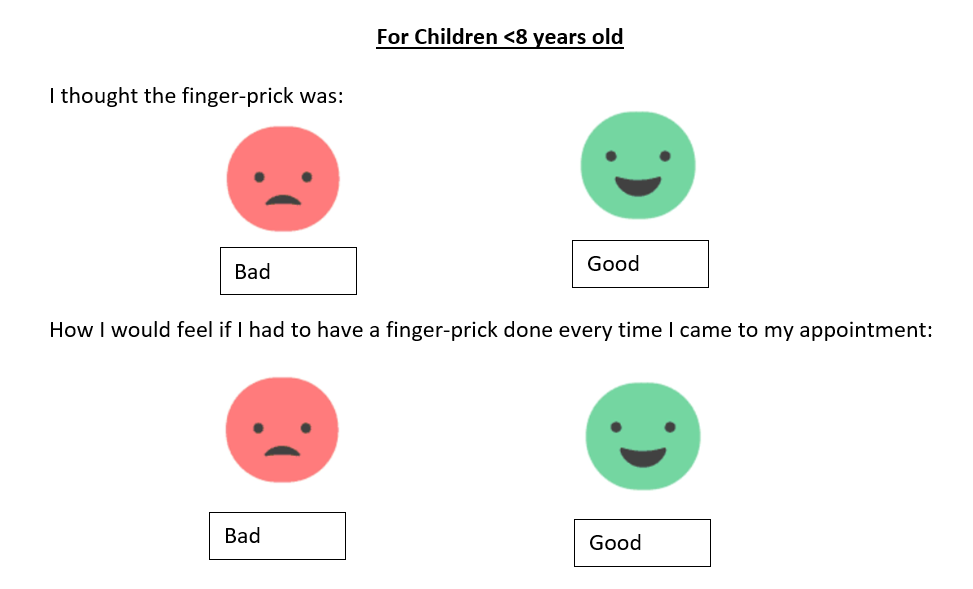
**

**Supplementary Figure 1B: LIKERT scale given to parents/ carers**

**
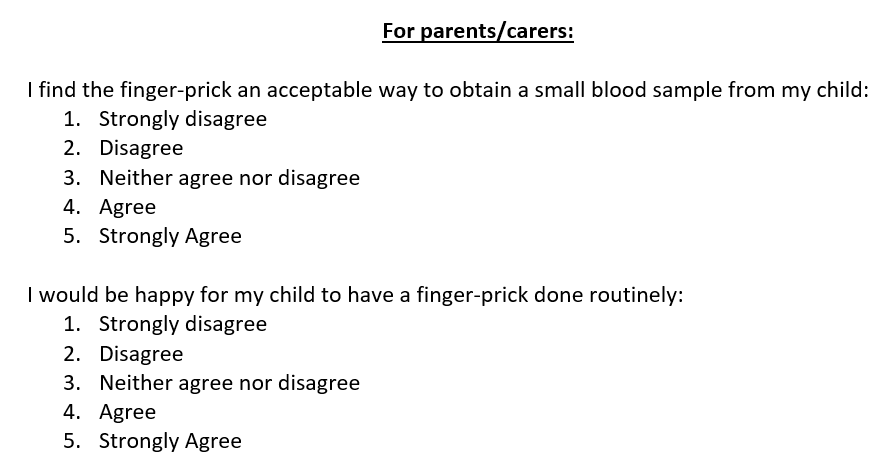
**

***Supplementary Figure 2 :*** *Likert scale used for assessing acceptability of point of care finger-prick blood test.* ***(A)*** *Questionnaire used for children (patients)* ***(B)*** *Questionnaire used for parents/ carers.*

**Supplementary Figure 3:**

***Supplementary Figure 3 :*** *Difference vs average: Bland-Altman of venous laboratory blood eosinophil counts vs capillary POC blood eosinophil counts. Bias: 0.046, SD of bias 0.052 and 95% limits of agreement was from -0.056 to 0.149. POC= Point of care*

**Supplementary Figure 4:**


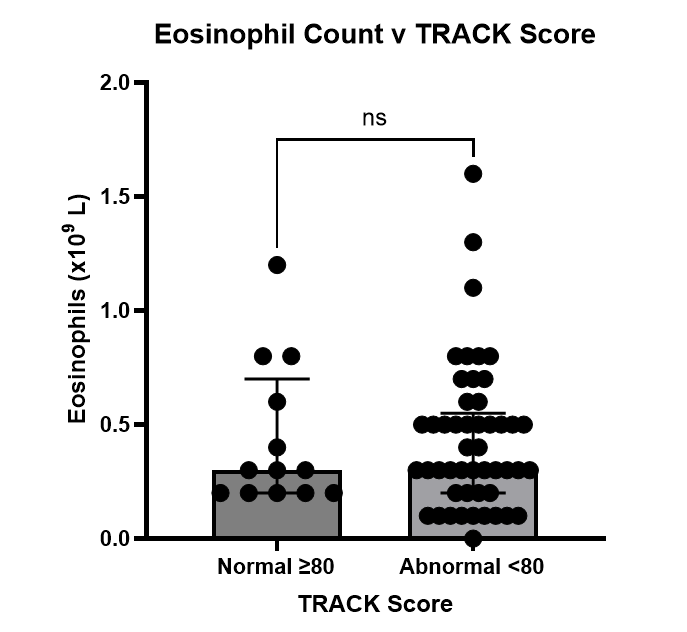


**A**

**B**

***Supplementary Figure 4A:*** *Relationships between blood eosinophil counts and* ***(A)*** *history of exacerbations and* ***(B)*** *TRACK score.*

***(A)*** *Blood eosinophil counts in children with no exacerbation (n=32) and at least one exacerbation (n=41) in the last 6 months. Median eosinophil counts were 0.3 x10^9^/L (IQR 0.1 x10^9^/L-0.7 x10^9^/L) in children (n=41) with no exacerbations during the previous 6 months, and 0.3 x10^9^/L (IQR 0.3 x10^9^/L- 0.5 x10^9^/L) in children with at least one exacerbation of wheeze (n=41). p value = 0.429.* ***(B)*** *Median eosinophils in children with TRACK score ≥80 (n=13) 0.3x10^9^/L (IQR 0.2 x10^9^/L – 0.7 x10^9^/L) and 0.3 x10^9^/L (IQR 0.2 x 0.6 x10^9^/L) in children with TRACK score <80 (n=49). p value = 0.989. Mann-Whitney U Test was performed. ns= not significant.*

*Error bars show interquartile range. Graph bar shows median. Individual participant data plotted as scatter plots. TRACK= Test for respiratory and asthma control in kids (TRACK): a caregiver-completed questionnaire for preschool-aged children*

**Supplementary Figure 5:**


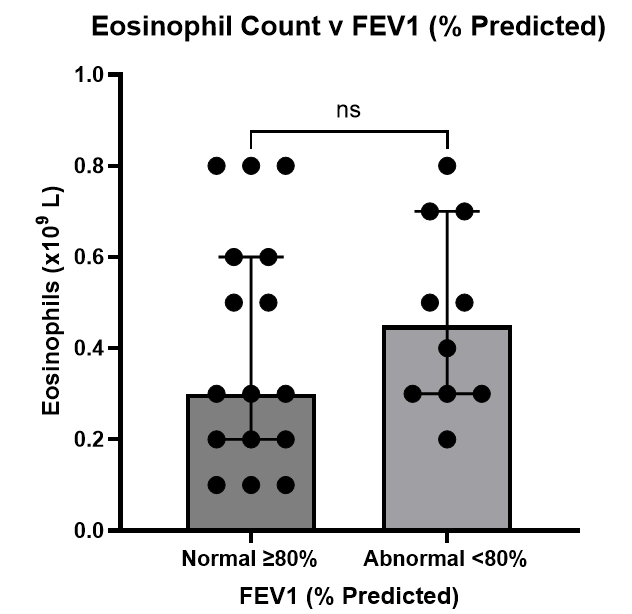


**A**

**B**

**C**

**D**

***Supplementary Figure 5:*** *Comparison between* ***(A)*** *age and ability to perform lung functions tests (FOT vs spirometry),* ***(B)*** *blood eosinophil counts in children with normal and abnormal spirometry. Relationships between blood eosinophil count and* ***(C)*** *FEV1 absolute and* ***(D)*** *FEV1 % predicted.*

***(A)*** *Median age of children who were able to perform FOT (n=34) was 4.25 years (IQR 3.7-5.15 years) compared to 5.45 years in children who were able to only perform spirometry (n=26) (IQR 4.9-5.73 years). P value = <0.0001* ***(B)*** *Median eosinophil count in children (n=16) with normal (FEV1≥80%) spirometry was 0.3x10^9^L (IQR 0.2x10^9^L- 0.6x10^9^L) compared to 0.45 x10^9^L (IQR 0.3x10^9^L- 0.7x10^9^L) in the cohort (n=10) with abnormal (FEV1<80%) lung function (n=10). p value=0.3990. Mann-Whitney U Test was used. ns = non-significant **** p <0.0001. Error bars show interquartile range.* *Black line shows linear regression with 95% confidence interval boundaries. Graph bar shows median. Individual participant data plotted as scatter plots.*

***(C)*** *Correlation between blood eosinophil count and FEV1 absolute value (r= -0.1871, p= 0.36, n=26).*

***(D)*** *Correlation between blood eosinophil count and FEV1 % predicted value (r= -0.2529, p= 0.2125, n=26). Horizontal red line in* ***C*** *and* ***D*** *represents cut-off value for eosinophilia (>0.3x10^9^/L). Spearman’s correlation was performed.*

*FOT= Forced oscillation technique, FEV1= Forced expiratory volume in one second*

**Supplementary Figure 6: Eosinophil Count v Xtot and Rtot Post-Bronchodilator Change (Z Score)**

**B**

**A**

F

***Supplementary Figure 6: Relationship between blood eosinophils and FOT measurements.*** *Red horizontal line in* ***A*** *and* ***B*** *represents cut-off value for eosinophilia (>0.3x10^9^/L). Non-parametric Spearman’s correlation was used.* ***(A)*** *Correlation between blood eosinophil count and post-bronchodilator change in reactance (Xtot z score change) (r= 0.495, p= 0.005, n=30)* *(B) Correlation between blood eosinophil count and post-bronchodilator change in resistance (Rtot z score change) (r= -0.312, p=0.09, n=30). Black solid line shows linear regression with 95% confidence interval boundaries.*

*FOT= Forced oscillation technique, Xtot= Total reactance, Rtot= Total resistance*

**Supplementary Figure 7A:** **Receiver operator characteristic** (**ROC) curve for eosinophil counts**


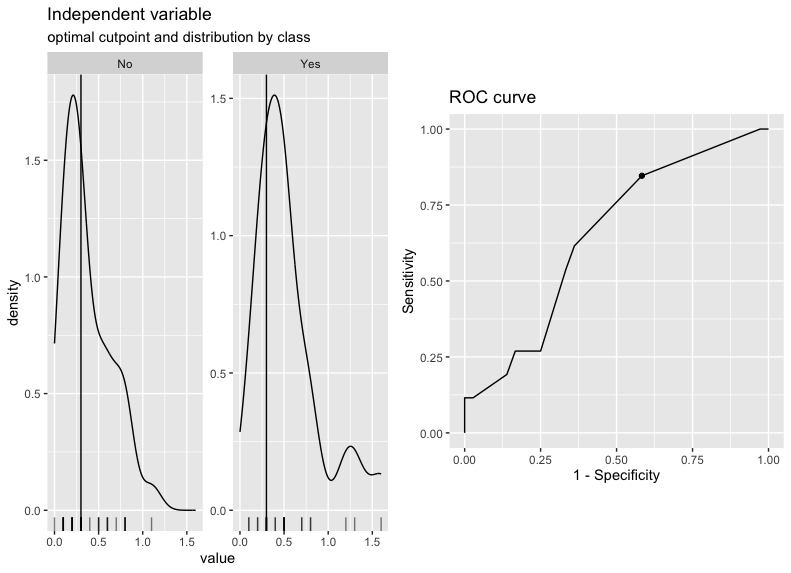


**Figure 7B: ROC Curve for eosinophil %**


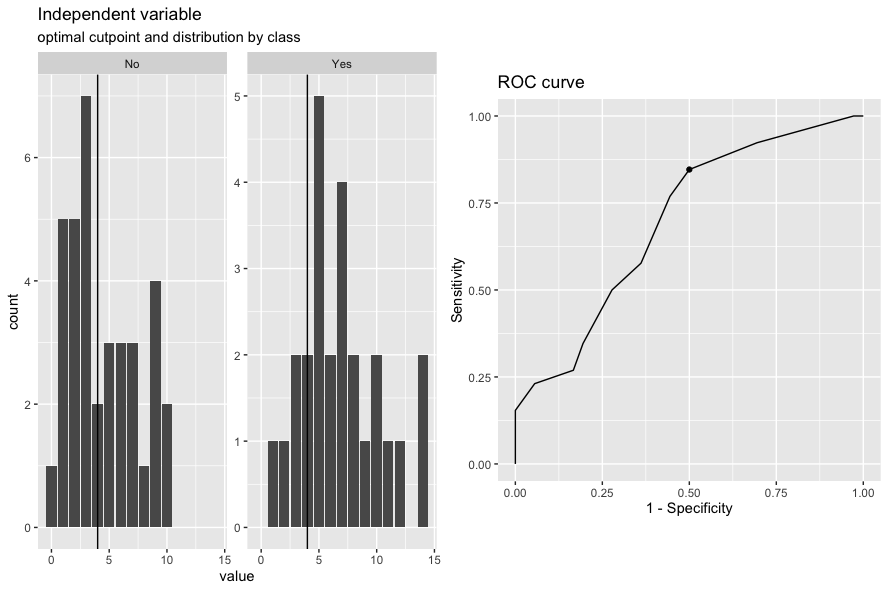


***Supplementary Figure 7:*** *Receiver operator characteristic curves* ***(A)*** *Eosinophil counts* ***(B)*** *Eosinophil %.*

**Supplementary Table 1: Comparison of absolute eosinophil count or percentage as a single biomarker vs combination with symptom score to predict wheeze attacks**

|  | **Area under Curve (AUC)** | **Optimal cut-off point** | **Accuracy** | **Sensitivity** | **Specificity** |
| --- | --- | --- | --- | --- | --- |
| **Eosinophil %** | 0.695 | 4% | 0.6452 | 0.8462 | 0.5 |
| **Eosinophil count** | 0.648 | 0.3x10^9^/L | 0.5968 | 0.8462 | 0.4167 |
| **Combined eosinophil % & TRACK score** | 0.72 | 4% and TRACK <75 | 0.71 | 0.69 | 0.73 |

**Supplementary Table 1:** TRACK= Test for respiratory and asthma control in kids (TRACK): a caregiver-completed questionnaire for preschool-aged children
